# Effects of Irrigation with Different Sources of Water on Growth, Yield and Essential Oil Compounds in Oregano

**DOI:** 10.3390/plants9111618

**Published:** 2020-11-20

**Authors:** Giuseppe Virga, Leo Sabatino, Mario Licata, Teresa Tuttolomondo, Claudio Leto, Salvatore La Bella

**Affiliations:** 1 Research Consortium for the Development of Innovative Agro-Environmental Systems (Corissia), Via della Libertà 203, 90143 Palermo, Italy; giuseppe.virga@corissia.it (G.V.); claudio.leto@unipa.it (C.L.); 2 Department of Agricultural, Food and Forest Sciences, Università Degli Studi di Palermo, Viale delle Scienze 13, Building 4, 90128 Palermo, Italy; leo.sabatino@unipa.it (L.S.); salvatore.labella@unipa.it (S.L.B.)

**Keywords:** aromatic species, constructed wetland, nonconventional water, irrigation, sustainability

## Abstract

Aromatic plants can benefit from the use of treated wastewater to satisfy their water requirements, but the effects on the essential oil yield and quality need an assessment. The aims of this study were to assess the effects of freshwater and treated wastewater obtained from a Sicilian (Italy) pilot-scale horizontal subsurface flow constructed wetland system on plant growth and yield, essential oil yield and composition of oregano (*Origanum vulgare* ssp. *hirtum* (Link) Ietswaart) and soil characteristics. The system had a total surface area of 100 m^2^ and was planted with giant reed and umbrella sedge. An experimental open field of oregano was set up close to the system. Two years and two different sources of irrigation water were tested in a split-plot design for a two-factor experiment. Treated wastewater was characterized by higher values of mineral and organic constituents than freshwater. The results highlight that short-term irrigation with freshwater and treated wastewater, in both years, led to increased plant growth, dry weight and essential oil yield of oregano plants. However, it did not significantly affect the essential oil content and composition in comparison with the control. Furthermore, the year and source of irrigation water did not significantly vary the chemical composition of the soil. Our results suggest that treated wastewater can be considered an alternative to freshwater for the cultivation of oregano due to the fact that it does not greatly influence the yield quality and quantity of this species in the short-term.

## 1. Introduction

*Origanum vulgare* ssp. *hirtum* (Link) Ietswaart is an aromatic plant that grows wild in various areas of the Southern Mediterranean region [1], and it is cultivated mainly for the aromatic, flavor and medicinal properties of the aerial parts and essential oil (EO) [2,3,4,5,6,7,8].

This perennial shrub is extremely rich in EO (1.10–8.20%, *v*/*w*, depending on the habitat) and can exhibit oil concentration, which is approximately 10 times higher than other subspecies of *Origanum vulgare* [9,10]. Carvacrol and thymol represent the dominant components of the EO and, on the basis of their prevalence, two main chemotypes are commonly attributed to this subspecies: carvacrol-chemotype, typically found in Greece [11,12,13] and thymol-chemotype, widespread in Sicily (Italy) and other Mediterranean areas [5,14,15,16]. Literature highlights that EO quality and quantity are significantly influenced by cultivation practices [17,18,19,20,21,22], environmental conditions [23,24,25,26,27] and genetic aspects [2,27,28,29,30,31]. Previous studies have focused on assessing the effect of water stress and nitrogen fertilizer on plant growth and yield and on EO yield and quality [18,32,33,34,35]. A number of authors agree that water stress can determine a change in EO composition and yield and a decrease in plant growth due to negative effects on photosynthesis and transpiration, while an appropriate irrigation schedule can greatly increase biomass production and EO yield [32,33,34,35]. Other authors state that the use of nutrients contributes to increasing plant biomass production and, more specifically, that nitrogen (N) fertilizers can determine a significant increase in EO yield in plants grown both in pots [36] and in open field conditions [18,19]. However, there is no clear evidence of the effect of N on EO content. In Greece, for example, the authors [19] found no significant effects of N on oil concentration during the growing period, while a reduction in oil concentration following N fertilization was shown in a study carried out under greenhouse conditions in Germany [33]. With regard to EO composition, it was found that N did not influence the carvacrol and thymol concentrations in inflorescences while increasing carvacrol and decreasing thymol in leaves through the conversion of π-cymene in carvacrol [19]. Therefore, the optimal combination of water supply through irrigation and N availability can increase the production of plant biomass and EO in oregano; however, some aspects, such as rainfall levels and distribution, and the cost of irrigation water and N fertilizers, can be limiting.

In areas with prolonged water shortage, as is found in the Mediterranean region, the use of treated wastewater (TWW) for combined irrigation and fertilization represents a sustainable cultivation practice also for aromatic plants. Many studies have examined the effects of TWW on horticultural and open field crops, evaluating how TWW affects plant growth and soil characteristics both in the short and long-term period [37,38,39,40,41,42,43,44,45,46,47]. Little attention has been paid to the use of TWW for the cultivation of aromatic species [48,49,50,51], probably due to cultural reasons related to public acceptance and bad perception of TWW reuse in agriculture [52,53,54]. It was found [48] that TWW irrigation did not significantly affect the yield quantity and quality and the essential oil (EO) composition in oregano and rosemary (*Rosmarinus officinalis* L.) in comparison with potable water irrigation. It was also demonstrated [49] that TWW determined a significant increase in EO yields in lemon verbena (*Aloysia citriodora* Palau) and a change in the main compounds of the EO in the species. In another study [50], it was observed that TWW irrigation increased EO yields in basil (*Ocimum basilicum* L.). In Egypt, five aromatic species were monitored for two years in order to evaluate the effect of TWW on EO quantity and quality, and it was reported that TWW irrigation of geranium (*Pelargonium graveolens* L’Hér) and fennel (*Foeniculum vulgare* Mill.) plants led to an increment in EO concentration, but to a reduction in peppermint (*Mentha piperita* L.) and sweet marjoram (*Origanum majorana* L.) plants [51]. It is well-known that the major risk associated with the use of TWW is the possible contamination of edible plants due to pathogen accumulation [43,44,45,46]. Therefore, wastewater (WW) must be treated and purified with single or combined treatment systems. One of the most interesting eco-technologies in agriculture with applications from urban to agricultural and industrial WW are constructed wetland systems (CWs) [41]. However, the use of TWW obtained from CWs for the irrigation of aromatic species is not documented in the literature.

In Sicily, oregano is usually grown without the use of irrigation. The species tends, in fact, to complete the main growth stages during spring, exploiting the water accumulated in the soil during autumn, winter and spring rainfall. However, irrigation can be required in certain conditions, when, for example, rainfall is not uniformly distributed. As natural water resources are often insufficient to carry out this practice, the use of nonconventional water such as TWW can be of considerable interest.

The main aims of the paper were to assess the effects of irrigation using TWW from a pilot horizontal subsurface flow system constructed wetland (HSSFs CW), compared to irrigation with FW, on (i) morphological and production characteristics of oregano, (ii) EO yield and quality, (iii) chemical soil characteristics.

## 2. Results and Discussion

### 2.1. Rainfall and Temperature Trends in the Experimental Site

**Figure 1:**
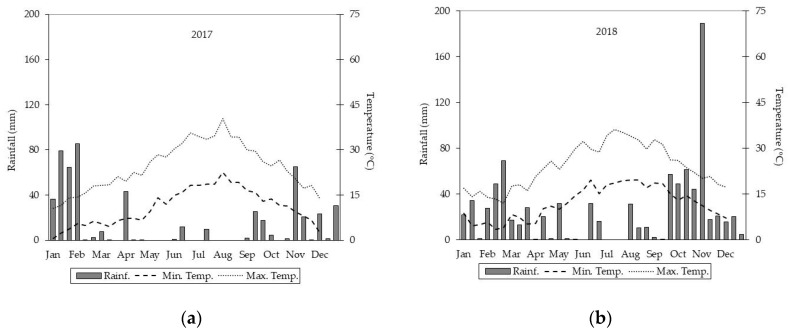
Rainfall and temperature trends during the test period. Graph (**a**) refers to 2017, while graph (**b**) refers to 2018.

In the test site, rainfall was far higher in 2018 than in 2017. Rainfall levels were, in fact, 534 mm and 894 mm for 2017 and 2018, respectively (Figure 1).

When observing all the months, the most significant levels of rainfall were recorded in the first 10-day period of November 2018 (188 mm). However, considering the period of greatest water requirement for plant growth (March to May) in the two years, rainfall levels showed a different trend (51.60 mm in 2017; 112.20 mm in 2018). In this period, rainfall levels were well distributed throughout the growing season in 2018, and this permitted an increase in soil moisture levels for a longer period compared to the 2017 growing season. Mean minimum and maximum temperatures in 2017 and 2018 were similar and consistent with the ten-year average temperature (16.0 °C).

Oregano plants did not show any frost damage during the winter months despite minimum temperatures falling below 3 °C in each growing season. Furthermore, heat damage was recorded in the plants when maximum daily temperatures rose over 35 °C, and rainfall was absent, confirming the high drought tolerance of oregano.

### 2.2. Freshwater and Treated Wastewater Characteristics

The main chemical and microbiological characteristics of FW and TWW used in this study are shown in Table 1.

The average values of the chemical and microbiological parameters at the outlet of the pilot HSSFs CW were not all within the threshold values established for TWW reuse for irrigation purposes by the Italian Ministerial Decree 152/2006. The age (old) and hydraulic conditions of the system affected, in fact, the removal of some parameters such as TSS and TP. Furthermore, the concentration levels of *E. coli* often exceeded legal limits. When comparing the FW and TWW concentrations during the test period, we observed great differences for most parameters. The lowest variations in nutrients and salts between FW and TWW were determined in spring and summer when the two macrophytes in the system grew fast and contributed significantly to WW treatment in terms of pollutant removal efficiency due to filtering, oxygen release and root exudates and uptake. The N level in TWW was much higher than that of FW during all sampling campaigns. This means that TWW represents an important source of N despite the removal processes carried out by plants, microorganisms and the medium in the pilot HSSFs CW. However, if we focus on the alkali metal levels in TWW in the study, we can affirm that TWW can be considered a source of macro and micronutrients, in accordance with study references [40,41,42,43,44]. Water quality was monitored using guidelines produced by the Food and Agriculture Organization of the United Nations [55] (Table 2).

When observing nutrient concentrations in the effluent of the HSSFs CW, we noted that the average levels of ammonium were high in the TWW and could determine increasing problems in plants and soil when used in irrigation. Average Na concentrations were found to cause increasing problems in irrigation, while that of Cl did not cause either minor or severe problems when used for irrigation purposes. Finally, average concentrations of EC in FW (0.26 dS m^−1^) and TWW (0.60 dS m^−1^) were found to be lower than those of the threshold values in the guidelines and were not critical for oregano growth.

### 2.3. Effects of Year and Source of Irrigation Water on Morphological and Yield Parameters of Oregano

**Figure 2:**
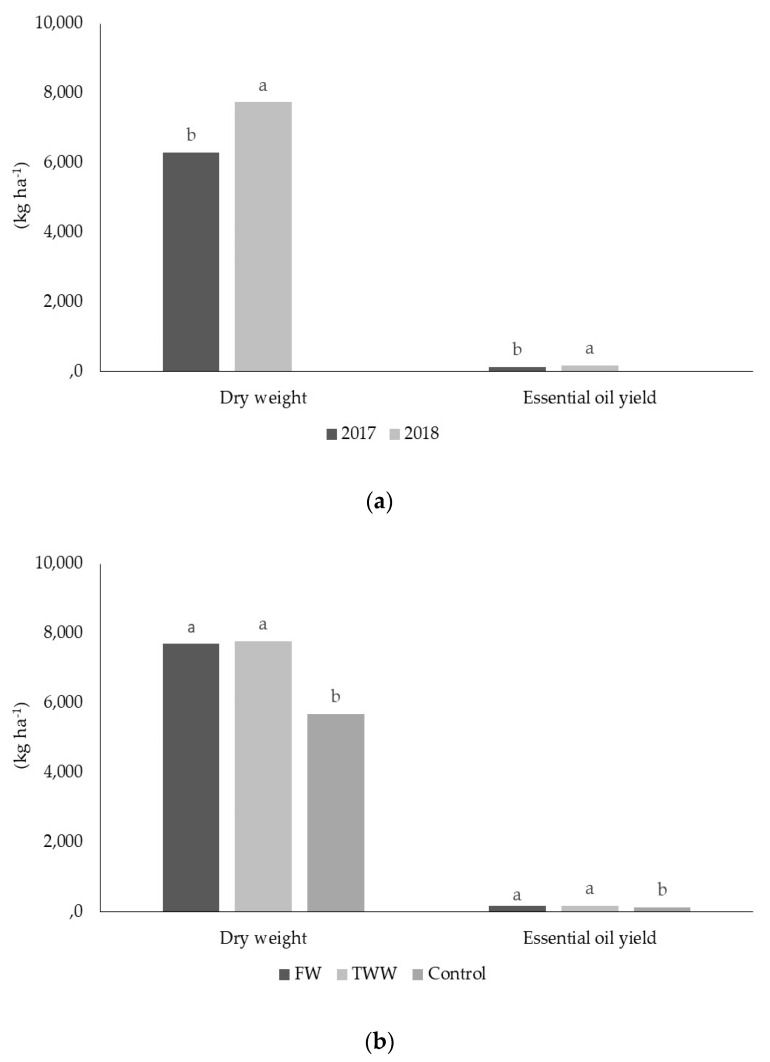
Effects of the main factors on dry weight and essential oil yield. Graph (**a**) refers to effect of the year; graph (**b**) refers to effect of source of irrigation water.

Data regarding the morphological and yield parameters of oregano plants, under the influence of year and source of irrigation water, are shown in Table 3 and Figure 2.

Year determined significant differences in plant height and the total number of branches per plant. In contrast, irrigation water exerted a significant effect on all morphological parameters. With regard to yield and yield components, it is worth noting that dry weight and EO yield were significantly influenced by year and irrigation water.

Results of analysis of variance also revealed that interactions between the main factors were not significant for all parameters in the study. When considering the morphological parameters, plant development was greater in FW- and TWW-irrigated plants than in unirrigated plants. Significant differences in plant height were found regarding year and source of irrigation water during the test period. In particular, plant height ranged from 59.05 cm (2017) to 64.04 cm (2018) on average. Furthermore, FW- and TWW-irrigated plants were taller than unirrigated plants on average. All the interactions between the main factors determined significant differences in plant height. The highest average values for plant height were obtained for year-by-FW and year-by-TWW interactions. On the contrary, the lowest average value for plant height was recorded for year-by-control interaction. Significant differences in plant diameter were found only with respect to the source of irrigation water. The highest average values of plant diameter were recorded for FW- and TWW-irrigated plants. Similar to plant height results, the highest values for plant diameter were obtained for year-by-FW and year-by-TWW interactions. Significant differences in the total number of branches per plant, however, were found regarding year and source of irrigation water. The highest average values for this morphological trait were obtained in 2018 and recorded for FW- and TWW-irrigated plants. Interactions between year and FW- and TWW treatments revealed the highest value for the total number of branches per plant.

Concerning yield parameters in the study, results were obtained in the second and third years of the study. The highest average values for dry weight and EO yield were recorded in 2018 compared to 2017. The source of irrigation water significantly affected dry weight and EO yield (Figure 2). More specifically, FW- and TWW-irrigated plants showed higher average values for dry weight and EO yield with respect to un-irrigated plants. Biomass production and EO yield did not differ significantly, comparing FW- and TWW irrigated plants. EO content was similar in irrigated and un-irrigated treatments.

Our results highlight the fact that FW- and TWW-irrigated plants showed greater growth in comparison with unirrigated plants in both years. This means that irrigation practice positively influenced the morphological development of oregano with respect to rainfed conditions. However, TWW was characterized by higher levels of macro and microelements, organic compounds and salts compared to FW. Despite this, irrigation with TWW did not compromise oregano plant growth. This fact could be explained by considering that, in particular, the amount of mineral and organic constituents in the TWW-irrigated plots was not significantly higher than that of the FW-irrigated plots.

Previous studies carried out on oregano, and other aromatic plants confirm our findings. In a study on oregano and rosemary [48], it was found that irrigation with treated effluent had no effect on overall plant development compared to irrigation with fresh water. In another study on the effect of irrigation intervals on morphological traits of oregano, the authors [56] found that reductions in plant height, stem diameter and other morphological parameters in longer irrigation intervals could be due to a decrease in cell enlargement and more leaf senescence or to a decrease in photosynthesis and could alter canopy structure using longer irrigation intervals. Furthermore, Leithy et al. [57] associated a decrease in plant growth of rosemary with a lower photosynthesis rate due to dry growing conditions. It was demonstrated [58] that the effect of reduced irrigation on the growth of lemongrass was due to the diminished availability of sufficient moisture in the rhizosphere and the lower absorption of nutrients by the plants. This concept is crucial and can also be related to other aromatic plants, such as oregano.

Literature highlights the importance of water as an essential factor for plant productivity [56,57,58,59]. It is well-known that water deficit can severely affect plant metabolism, determining a series of morphological and physiological changes that can reduce plant yield. When considering aromatic plants, a number of studies [32,33,34,35,56,60] underline the effect of water deficit on plant biomass and EO content and yield. In a study on EO response to water stress in Greek oregano populations, the authors [35] stated that water deficiency significantly decreased dry matter production and EO yield but significantly increased EO content. The same findings were found by Azizi et al. [33], who highlighted that water deficiency after the beginning of blooming induced and increased EO content and thus resulted in higher quality oregano herbage. It was also reported [34] that the dry weight of the above-ground plant parts and the total weight of the leaf and inflorescence significantly decreased in oregano under mild and moderate water stress, in comparison to those of plants grown under normal conditions. The same authors observed that EO content did not significantly change under water stress conditions in comparison with the control.

In our study, the main productive parameters did not differ significantly in both TWW- and FW-irrigated plants. This was in agreement with the findings of Bernstein et al. [48] and confirm the use of TWW as an alternative to FW for oregano cultivation in areas with prolonged water shortage. When analyzing EO content in various treatments, it was found to be greater in irrigated treatments, but the interaction with the year was not significant. This was in contrast with literature, which highlights that EO content and production can be stimulated by stressful environments [61]. In particular, it was reported [24] that, in response to water stress conditions and in order to limit transpiration rates, aromatic plants developed the ability to channel secondary metabolism into the production of EOs. It was documented [56] that, in case of stress, more secondary metabolites are produced in the plant cells, and EOs represent the product of respiratory catabolic processes, which increase under water deficit. It was also demonstrated [17,62] that water stress tends to reduce the leaf area of the plant, favoring a higher density of glandular trichomes producing EO on the leaf tissue; the amount of EO per unit of leaf tissue thereby increasing as a result. Our findings can be explained by observing the rainfall trends during the growth period in both years. It is reasonable to assume that rainfall greatly increased the moisture level of the soil; therefore, soil water availability of unirrigated plants was similar to that of irrigated-plants and no significant differences for EO content were found between the various treatments.

### 2.4. Effects of Year and Source of Irrigation Water on Composition of the Essential Oil

Data of the most representative EO compounds in response to the year and the source of irrigation water are shown in Table 4.

Twenty-nine compounds were identified in total for the oregano EO (Supplementary Tables S1 and S2).

Year and source of irrigation water did not alter the composition of the essential oil of the oregano plants throughout the entire study period. When comparing the two years, no significant differences were found for any EO compound. Furthermore, the percentage amounts of the most representative EO compounds were similar for FW-, TWW-irrigated plants and unirrigated plants. In the case of year and irrigation, thymol (ranging from 39.31% to 41.03%) was the main component of oregano EO, as it has been previously reported in many studies carried out in Sicily [5,14,16,17,24]. The second major constituent was γ-terpinene (ranging from 23.70% to 25.01%).

Our findings highlight that FW- and TWW-irrigation does not have any significant effect on the composition of oregano EO. This was partially in agreement with the literature. Previous studies conducted in other regions [33,34,48] show that water supply regimes do not cause significant changes in the composition of oregano EO. In a study carried out on Greek oregano [59], it was reported that significant differences for ρ-cymene content in inflorescences and leaves were found between irrigated and dry treatments. This was in contrast with our results and underlined the importance of water soil availability in the cultivation of oregano. Similar to the effects of year and source of irrigation water on morphological and yield parameters, it is plausible to claim that a severe lack of water availability in the soil could determine a significant change in the EO composition of oregano plants in comparison with good water availability.

### 2.5. Effects of Year and Source of Irrigation Water on Soil

The chemical characteristics of the FW-irrigated soil, TWW-irrigated soil and unirrigated soil are reported in Table 5.

The year and source of irrigation water did not lead to significant changes in the chemical composition of the soil during the study period.

No significant variations in topsoil pH of the FW- and TWW-irrigated soils were recorded in comparison with unirrigated soil. Our results were in accordance with findings in the literature. Bernstein et al. [48], in a 3-year study, found that the pH of the soil was similar in both potable-water-irrigated soil and secondary-effluent-irrigated soil. The same authors also reported that, at high pH values, the availability of nutrients to the plant roots could change due to alterations in the soluble compounds in the fertigation and soil solutions and to changes in ion adsorption of the soil complex. In our study, in particular, the non-significant response of the plants to the higher pH average value of TWW (7.67) could result from the short-term application of TWW in both years or from plant resistance, as shown in previous studies [48,50,63].

Despite the high EC level of TWW compared to FW, the EC of TWW-irrigated soils was not significantly different from that of FW-irrigated soils. Only a light accumulation of salts originating from TWW was apparent in the topsoil, despite the moderate percentage of clay in the soil structure. Furthermore, non-significant differences were found between FW-, TWW-irrigated soils and the control due to limited exposure to higher EC levels in TWW, in particular. For this reason, no signs of salinity damage were observed in the plants. Oregano is, in fact, sensitive to soil salinity; therefore, increasing salinity of the water causes a significant decrease in biomass and EO yields, as reported by Hancioglu et al. [64].

TOC increased with TWW irrigation, but no significant differences were found with respect to FW-irrigation and the control. It is reasonable to suppose that the effects of TWW irrigation on topsoil organic matter are highly correlated to the irrigation duration and the amount of organic compounds in the TWW. Thus, the greater the levels of TOC in TWW and the longer TWW is used for irrigation, the more the topsoil TOC increases [44].

With regard to N, P and K levels in the soil, it is evident that nutrient accumulation in the topsoil can be linked to the original levels in FW and TWW, but these soil levels tend to change due to irrigation duration and the main actions conducted by plants and soil microorganisms, in particular. In this study, FW and TWW irrigation increased N, P and K levels in the topsoil; however, differences found between the main factors were not significant due to the short-term irrigation period in both years. Similar results were also found by Bernstein et al. [48], who state that variability in plant response to N amounts and to the relationship between N forms exists within and between aromatic species.

Regarding the alkali metals contained in the irrigation water, Na is of concern due to its negative effects on soil properties. It is well-known that an excess of Na in the soil can displace divalent cations, such as Ca and Mg, leading to soil structure deterioration [44]. In our research, despite higher Na concentrations in TWW compared to FW, we did not observe significant differences between the main treatments. Only Na levels in TWW-irrigated soil were found to be higher than in FW-irrigated soil and the control. The short duration did not lead to significant accumulation of Na in the topsoil or a displacement of Ca and Mg in the structural aggregates of the soil. Finally, it is worth noting that average SAR values, as shown in Table 1, remained below values that could negatively affect soil properties (SAR > 10). This allowed the plant roots to grow well and not suffer damage due to excess Na concentrations.

## 3. Materials and Methods

### 3.1. Experimental Site

Trials were carried out in the experimental area of the pilot HSSFs CW in Raffadali (Italy), the South-West of Sicily (37°59’56”40 N–13°16’50”16 E, 740 m a.s.l.), during the growing seasons of 2016–2018. An experimental open field of oregano was set up close to the pilot HSSFs CW. According to the Köppen–Geiger climate classification [65], the study location is characterized by a warm temperate climate with dry summers. Average annual rainfall is approximately 650 mm, mainly distributed between October and April. With reference to time series 1982–2012, the annual average temperature was 17.50 °C, the average maximum temperature was 23.50 °C, and the average minimum temperature was 11.20 °C. The soil type in the area is clay loam (40% sand, 21% silt and 39% clay), and it is classified as Brown Soil-Brown Leached Soil-Regosols Soil (USDA classification).

### 3.2. Pilot HSSFs CW

**Figure 3:**
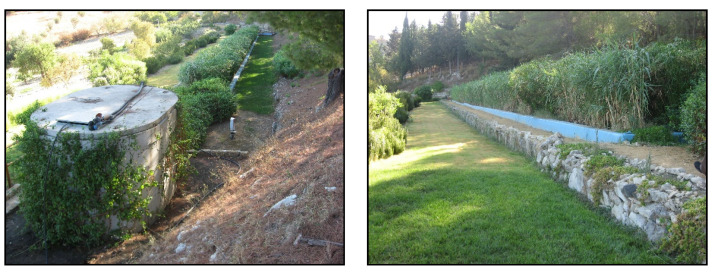
An overview of the horizontal subsurface flow system constructed wetland (HSSFs CW).

The pilot HSSFs CW is located in an open urban park vegetated with turfgrass and ornamental plant species (Figure 3).

The system consists of two independent units in parallel (50 m-long and 1 m-wide) and provides a total surface area of 100 m^2^. The two units are separately planted with giant reed (*Arundo donax* L.) and umbrella sedge (*Cyperus alternifolius* L.). The functional and technical characteristics of the system were previously described by the authors [39].

During the test period, the system was fed with urban WW from the wastewater treatment plant (WWTP) in the town. It was designed to receive a total of 6 m^3^ of WW per day. TWW was evenly distributed in the units, and the pumping of TWW was continuous throughout the day without variations in time. The outflow TWW flowed downhill into four storage tanks of 5 m^3^ each, the last of the tanks was connected to a drip system for irrigation purposes. The two planted units operated under the same hydraulic conditions. Hydraulic loading rate (HLR) was 6 cm day^−1,^ and hydraulic retention time (HRT) was 8.3 per day.

**Figure 4:**
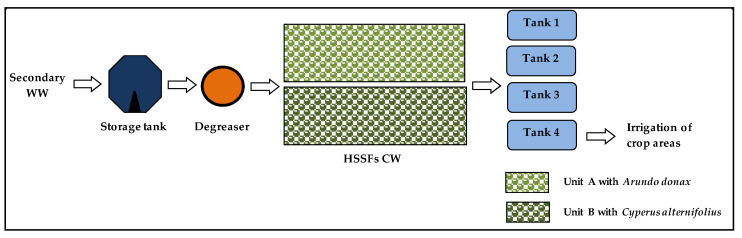
Layout of the HSSFs CW.

The layout of the HSSFs CW for the treatment of the wastewater is shown in Figure 4.

### 3.3. Urban Wastewater Analyses

In HSSFs CW, sampling campaigns were carried out monthly during the period April–September for each year. A total of 48 samples were taken and analyzed. A liter of wastewater was collected at the inlet (0 m) and the outlet (50 m) of each unit during each sampling. The influent and effluent samples were instantaneous samples. Sampling always occurred at the same time as 9:00 a.m. per day. The pH value (± 0.01 pH), electrical conductivity (EC) (0.05% of value), temperature (T), and dissolved oxygen levels (DO) (0.05% of value) were determined directly on-site at the time of sampling using a portable universal meter (Multiline WTW P4), in compliance with the calibration protocol for each of the four parameters. Total suspended solids (TSS), biochemical oxygen demand (BOD_5_), chemical oxygen demand (COD), total Kjeldahl nitrogen (TKN), ammonia nitrogen (NH_4_-N), total phosphorus (TP), sodium (Na), potassium (K), calcium (Ca), magnesium (Mg) and chloride (Cl) levels were determined in the laboratory [66]. Total coliform (TC), fecal coliform (FC), fecal streptococci (FS), *Escherichia coli* (*E*. *coli*) and *Salmonella* spp. levels were determined by membrane filter technique, based on standard methods for water testing [67].

### 3.4. Oregano Experimental Field and Main Cultivation Practices

An oregano biotype gathered from Monreale (Italy) (38°4’51”96 N–13°17’21”84 E, 310 m a.s.l.) and classified as *Origanum vulgare* L. ssp. *hirtum* (Link) Ietswaart was used for the tests. This biotype was named “Monrealease” and showed an erect growth, green oval leaves and flowers with a white corolla. In November 2015, the herbaceous cuttings were taken from mother plants and placed in a cold greenhouse for rooting. In the first decade of January 2016, the rooted cuttings were manually transplanted into the experimental field. Plant density was 2.6 plants m^−2^. The distance between rows and within rows was 1.50 m and 0.25 m, respectively. The single plot size was 45 m^2^.

The experimental field was equipped with two drip irrigation systems, one for each source of irrigation water used in the study (FW and TWW). Self-compensating drippers were used for the tests and were located 0.25 m apart. The use of this type of dripper guaranteed a constant flow rate and ensured the distribution of the precise amount of water and nutrients into the plot. In both years and for FW- and TWW-irrigated plots, 2 drip irrigation events were effectuated, supplying 400 m^3^ ha^−1^ of water during each event. The irrigation events were planned during the maximum plant growth stages and were carried out on a monthly basis from the third 10-day period of March, both in 2017 and 2018. In contrast, unirrigated plants exploited rainfall only. Before transplanting, the experimental field received a total of 30 kg ha^−1^ N and 90 kg ha^−1^ P_2_O_5_. During the two growing seasons, all irrigated and unirrigated treatments received 60 kg N ha^−1^: a commonly used fertilization program for oregano in Sicily.

Weeds were controlled mechanically, and no pesticides were used in both years. Harvest was carried out when most plants were at the full blooming stage. In this stage, plants were cut at approximately 0.10 m above the ground, in accordance with conventional agronomic practices. In 2017, above-ground plant parts were manually harvested on 10th June for unirrigated plants and on 25th June for FW- and TWW-irrigated plants. In 2018, harvesting dates were 8th June for unirrigated plants and 22nd June for FW- and TWW-irrigated plants.

### 3.5. Morphological and Productive Parameters

Plant height was measured from the soil surface to the tip of the tallest flowering stem at the full blooming stage. The fresh matter weight of above-ground plant parts (data not shown) was determined by harvesting ten randomly selected plants from the center rows of every single plot to avoid any border effect. The harvested plant material was then dried in an oven at 65 °C for 48 h until it reached a constant weight. The plant dry matter weight was then calculated. The proportion of inflorescences, leaves and stems were separately determined by weighing these components with a digital balance. Plant diameter and the total number of branches per plant were also determined in the laboratory. For all morphological and yield parameters, the second and third growing seasons only were considered due to the fact that in the first growing season, the plants were young, and the main yields were not significant.

### 3.6. Isolation, Analyses of the Essential Oils and Identification of Components

Essential oil content was obtained by hydrodistillation of air-dried plant material (50–100 g) for 3 h in accordance with international guidelines [68]. EO content was determined in the second and third years since herbage production in the first year was low. EO was dried on anhydrous sodium sulfate and stored under N_2_ until required. Determination of EO composition was performed by gas chromatography–mass spectrometry (GC–MS) analyses. GC analyses were run on a Shimadzu gas chromatograph, Model 17-A (Shimadzu Corporation, Duisburg, Germany), equipped with a flame ionization detector (FID). Analytical conditions: SPB-5 capillary column (15 m × 0.10 mm × 0.15 μm), helium as carrier gas (1 mL min^−1^). Injection in split mode (1:200), injected volume 1 μL (4% essential oil/CH2Cl2 *v*/*v*), injector and detector temperature 250 and 280 °C, respectively. The oven temperature was held at 60 °C for 1 min, then programmed from 60 to 280 °C at 10 °C min^−1^, then 280 °C for 1 min. Percentages of compounds were determined from their peak areas. GC–MS was carried out in the fast mode on a Shimadzu GC–MS model. GCMS-QP5050A, with the same analytical conditions used for GC-FID. Ionization voltage 70 eV, electron multiplier 900 V, ion source temperature 180 °C. Mass spectra data were acquired in the scan mode in *m*/*z* range 40–400. The same oil solutions (1 μL) were injected in the split mode (1:96). All analyses were carried out in triplicate. Identification of the compounds was based on GC retention index (relative to C9-C22 n-alkanes on the SPB-5 column), computer matching of spectral MS data with those from the National Institute of Standards and Technology (NIST) MS libraries [69], comparison of the fragmentation patterns with those reported in the literature [70] and, whenever possible, co-injections with authentic samples. Pure standards were purchased from Aldrich Chemical Co., Extrasynthese, France and FlukaChemie AG, Switzerland. All the analyses were carried out at the Corissia Research Center in Palermo (Italy).

### 3.7. Soil Analyses

PH, electrical conductivity (EC), total organic carbon (TOC), total nitrogen (TKN), assimilable phosphorus (P), assimilable potassium (K), active total calcareous (CaCO_3_), magnesium (Mg) and sodium (Na) content were the main soil parameters analyzed in the study. Sampling was carried out in a soil layer ranging from 0 to 40 cm, having oregano plants an average depth of the root system equal to 35 cm. Four sampling spots per plot were combined for the plot sample. Soil samples were subsequently air-dried, ground, sieved through a 2-mm sieve screen and then analyzed for chemical and physical characteristics. The samples were analyzed for pH and EC in the ratio of 1:2 dry soil: water extract; pH was determined with a calibrated pH-meter, EC with a calibrated conductivimeter, TOC with the Walkley and Black method [71], TKN by the Kjeldahl procedure [72], assimilable P by the Olsen method [73] and total calcareous using the Drouineau method [74]. K (± 0.08, ppm), Mg and Na contents were determined by atomic absorption spectrophotometer. All the analyses were carried out at the Corissia Research Center of Palermo.

### 3.8. Weather Data

A weather station belonging to the agrometeorological information service of the Sicilian Government [75] was used in this study to collect climate data. It was located close to the experimental site and was characterized by instruments and sensors for the measurement of the main climate factors, such as air temperature, global solar radiation, leaf wetness, rainfall, relative humidity and wind speed. In particular, the station provided an MTX datalogger (model WST1800, Padova, Italy), which was used for the collection of daily minimum and maximum air temperatures and total 10-day rainfall data.

### 3.9. Experimental Design and Statistical Analyses

A split-plot design for a two-factor experiment was used with three replications. The main plot factor was the year (Y) with two treatment levels: Y_1_ (2017); Y_2_ (2018). The subplot factor was irrigation water (IW) with three treatment levels: IW_1_ (FW), IW_2_ (TWW), IW_3_ (rainfed), as control. Three replications for each sub-plot were considered. Statistical analyses were performed using the package MINITAB 17 for Windows. Data were compared using analysis of variance. The difference between means was carried out using Tukey’s test. For FW and TWW, all the representative values were presented using mean ± standard error calculation.

## 4. Conclusions

The results of this study highlight that irrigation with treated wastewater represents a sustainable practice for growing oregano in areas with prolonged water shortages. In the short-term, irrigation with treated wastewater does not affect plant growth and production or the essential oil content and yield in comparison with freshwater. Furthermore, the percentage of the main compounds such as thymol and γ-terpinene remains unaffected. When also considering the rainfed treatment, irrigation with freshwater and treated wastewater affects the biomass and EO yields, but it does not significantly influence the essential oil quality and quantity of oregano plants.

As the quality of aromatic plants is determined both by the content and composition of its essential oil, it is evident that treated wastewater obtained by eco-technologies, such as constructed wetland systems, can be a useful tool both to save fresh water and to obtain promising agronomic results in qualitative and quantitative terms.

In this study, the chemical characteristics of soil were not significantly affected by irrigation treatments, and the mineral and organic constituents provided by treated wastewater, in particular, did not negatively modify the soil fertility and, thus, damage the plants.

However, further research is required to assess the effect of irrigation with treated wastewater on morphological and productive characteristics of oregano, also in the long term, to avoid the possible accumulation of mineral and organic compounds in the topsoil, provoking salt injury to the plants.

## Figures and Tables

**Table 1:** Main chemical and microbiological characteristics of the freshwater and treated wastewater that were used during oregano irrigation. Average values (± standard error) are shown (*n = 6*).

Parameter	FW	TWW	Threshold Values (Italian Ministerial Decree No. 152/2006)
pH	7.11 ± 0.11	7.95 ± 0.75	6–9.5
EC (μS cm^−1^)	264.01 ± 0.13	510 ± 0.12	3000
BOD_5_ (mg L^−1^)	1.77 ± 0.03	12.12 ± 0.09	20
COD (mg L^−1^)	2.34 ± 0.05	14.32 ± 0.10	100
TSS (mg L^−1^)	Not detected	13.01 ± 0.13	10
TP (mg L^−1^)	0.87 ± 0.64	4.63 ± 0.08	2
NO3-N (mg L^−1^)	0.29 ± 0.32	4.90 ± 0.21	-
TKN (mg L^−1^)	1.98 ± 1.01	14.90 ± 1.13	15
N-NH_4_ (mg L^−1^)	0.82 ± 1.12	9.62 ± 0.24	2
Ca (mg L^−1^)	25.11 ± 0.32	61.12 ± 0.71	-
Cl (mg L^−1^)	14.12 ± 0.26	121.01 ± 3.24	250
K (mg L^−1^)	3.01 ± 1.13	75.77 ± 0.99	-
Mg (mg L-1)	11.99 ± 1.34	22.11 ± 0.21	-
Na (mg L^−1^)	11.45 ± 0.18	137.11 ± 1.14	-
Heavy metals	Not detected	Not detected	-
Sodium adsorption ratio (SAR) (meq L^−1^)	0.32 ± 0.22	2.70 ± 0.14	-
*Escherichia coli* (CFU 100 mL^−1^)	0.78 ± 0.01	2.11 ± 0.04	10 (80% of samples) and 100 (maximum value point)
TC (CFU 100 mL^−1^)	0.98 ± 0.07	3.11 ± 0.07	-
FC (CFU 100 mL^−1^)	0.86 ± 0.02	3.37 ± 0.10	-
FS (CFU 100 mL^−1^)	0.91 ± 0.03	3.10 ± 0.01	-
*Salmonella* spp. (CFU 100 mL^−1^)	Absent	Absent	-

**Table 2:** General guidelines for the interpretation of water quality for crop irrigation [55].

Item	Minor Problems	Increasing Problems	Severe Problems
Salinity			
EC (water) (mmhos cm^−1^ or dS m^−1^)	<0.75	0.75–3.0	>3.0
Specific ion toxicity			
Na (meq L^−1^)	<3.0	3–9	>9
Cl (meq L^−1^)	<4.0	4–10	>10
B (meq L^−1^)	<0.75	0.75–2.0	>2.0
Miscellaneous effects			
N-NH_4_ (mg L^−1^)	<5	5–30	>30
NO_3_-N (mg L^−1^)	<5	5–30	>30
HCO_3_ (meq L^−1^)	<1.5	1.5–8.5	>8.5
pH	normal range 6.5–8.4		

**Table 3:** Morphological and yield characteristics of oregano plants in response to year and source of irrigation water during the study period.

Treatment	Plant Height (cm)	Plant Diameter (cm)	No. Branches (per Plant)	Dry Weight (t ha^−1^)	Flowers (% of Dry Weight)	Leaves (% of Dry Weight)	Stems (% of Dry Weight)	Essential Oil Content (%)	Essential Oil Yield (kg ha^−1^)
Year									
Y1	59.05 b	82.77 a	204.78 b	6.30 b	24.93 a	23.81 a	51.26 a	2.32 a	146.33 b
Y2	64.04 a	85.73 a	223.67 a	7.74 a	24.76 a	23.72 a	51.52 a	2.35 a	182.78 a
Irrigation water									
IW1	64.89 a	92.17 a	236.33 a	7.70 a	24.73 a	23.07 a	52.19 a	2.41 a	178.02 a
IW2	65.33 a	89.63 a	246.43 a	7.68 a	25.57 a	23.74 a	50.69 a	2.31 a	186.61 a
IW3	54.41 b	70.97 b	160.00 b	5.68 b	24.22 a	24.48 a	51.29 a	2.28 a	129.03 b
Year Irrigation water									
Y1 × IW1	63.13 ab	93.66 a	143.33 b	7.06 c	24.46 a	23.38 a	52.15 a	2.34 a	165.37 ab
Y1 × IW2	62.17 ab	88.33 a	228.33 a	7.16 bc	26.33 a	23.68 a	49.98 a	2.30 a	164.85 ab
Y1 × IW3	51.83 c	66.34 c	242.66 b	4.67 d	23.98 a	24.36 a	51.65 a	2.32 a	108.77 c
Y2 × IW1	63.61 a	90.67 a	176.66 b	8.33 a	25.00 a	22.77 a	52.23 a	2.48 a	207.85 a
Y2 × IW2	68.50 a	90.92 a	244.33 a	8.21 ab	24.81 a	23.79 a	51.39 a	2.33 a	191.20 ab
Y2 × IW3	57.00 bc	75.60 b	250.00 a	6.88 c	24.46 a	24.60 a	50.94 a	2.23 a	149.28 bc

Means followed by the same letter are not significantly different for *p* ≤ 0.05 according to Tukey’s test.

**Table 4:** Composition of the essential oil of oregano plants in response to year and source of irrigation water during the study period. Data on the most representative essential oil compounds are shown.

Treatment	*α*-Thujene (%)	*ß*-Myrcene (%)	*α*-Yerpinene (%)	*ρ*-Cymene (%)	*γ*-Terpinene (%)	Carvacrol Methyl Ether (%)	Thymol (%)
Year							
Y1	2.08 a	2.96 a	4.10 a	8.05 a	24.32 a	2.77 a	40.01 a
Y2	2.16 a	2.97 a	4.15 a	8.12 a	24.28 a	2.87 a	39.97 a
Irrigation water							
IW1	2.13 a	2.95 a	4.18 a	8.03 a	24.35 a	2.78 a	39.85 a
IW2	2.08 a	3.01 a	4.07 a	8.13 a	24.33 a	2.87 a	39.98 a
IW3	2.16 a	2.96 a	4.13 a	8.10 a	24.23 a	2.80 a	40.15 a
Year × Irrigation water							
Y1 × IW1	2.16 a	2.93 a	4.13 a	7.93 a	24.37 a	2.70 a	39.80 a
Y1 × IW2	1.97 a	3.01 a	4.10 a	8.20 a	24.23 a	2.80 a	39.83 a
Y1 × IW3	2.13 a	2.97 a	4.07 a	8.03 a	24.37 a	2.80 a	40.40 a
Y2 × IW1	2.10 a	2.97 a	4.23 a	8.13 a	24.33 a	2.87 a	39.90 a
Y2 × IW2	2.20 a	3.01 a	4.03 a	8.06 a	24.43 a	2.93 a	40.13 a
Y2 × IW3	2.10 a	2.97 a	4.20 a	8.17 a	24.10 a	2.80 a	39.90 a

Means followed by the same letter are not significantly different for *p* ≤ 0.05 according to Tukey’s test.

**Table 5:** Main chemical characteristics of soil in response to year and source of irrigation water during the study period.

Treatment	pH	EC (μS cm^−1^)	TOC (g kg^−1^)	TKN (g kg^−1^)	P (mg kg^−1^)	Total CaCO_3_ (g kg^−1^)	Na (ppm)	K (ppm)	Mg (ppm)
Year									
Y1	7.62 a	195.72 a	7.67 a	1.26 a	30.71 a	1.33 a	90.56 a	542.30 a	633.60 a
Y2	7.65 a	199.12 a	7.72 a	1.25 a	31.01 a	1.34 a	90.90 a	543.60 a	634.68 a
Irrigation water									
IW_1_	7.63 a	195.95 a	7.64 a	1.25 a	30.51 a	1.34 a	90.66 a	542.77 a	632.78 a
IW_2_	7.67 a	203.89 a	7.72 a	1.29 a	31.20 a	1.36 a	91.35 a	546.42 a	638.80 a
IW_3_	7.61 a	191.12 a	7.67 a	1.22 a	30.72 a	1.32 a	89.98 a	541.64 a	632.31 a
Year x Irrigation water									
Y1 × IW_1_	7.62 a	194.83 a	7.64 a	1.24 a	30.35 a	1.33 a	90.30 a	541.18 a	631.78 a
Y1 × IW_2_	7.63 a	199.80 a	7.73 a	1.29 a	31.24 a	1.35 a	91.60 a	544.22 a	636.82 a
Y1 × IW_3_	7.60 a	193.80 a	7.62 a	1.24 a	30.53 a	1.33 a	89.56 a	540.52 a	632.15 a
Y2 × IW_1_	7.64 a	195.25 a	7.65 a	1.23 a	30.67 a	1.35 a	91.04 a	544.36 a	633.78 a
Y2 × IW_2_	7.65 a	199.97 a	7.77 a	1.30 a	31.55 a	1.36 a	91.78 a	549.66 a	638.77 a
Y3 × IW_3_	7.62 a	193.82 a	7.66 a	1.24 a	30.33 a	1.32 a	89.87 a	542.77 a	633.48 a

Means followed by the same letter are not significantly different for *p* ≤ 0.05 according to Tukey’s test; n.s.: not significant.

## Supplementary Materials

The following are available online at https://www.mdpi.com/2223-7747/9/11/1618/s1, Table S1: Effect of the year on the chemical composition of essential oregano oil; Table S2: Effect of irrigation water on the chemical composition of oregano essential oil.
