# Supplementary material for: Effects of Irrigation with Different Sources of Water on Growth, Yield and Essential Oil Compounds in Oregano
Source: Plants (Basel). 2020 Nov 20;9(11):1618. doi: 10.3390/plants9111618 (PMC7699947; doi:10.3390/plants9111618)
Supplement: Supplementary file 1 [file plants-09-01618-s001.pdf]

Table S1. Effect of year on chemical composition of oregano essential oil. Average values (± standard deviation) are shown (*n* = 9).

Compound	2017	2018
α-Thujene	2.10 ± 0.13	2.21 ± 0.13
α-Pinene	1.00 ± 0.12	1.11 ± 0.13
Sabinene	0.21 ± 0.07	0.22 ± 0.04
ß-Pinene	0.52 ± 0.08	0.61 ± 0.07
ß-Myrcene	3.01 ± 0.13	3.02 ± 0.13
α-Phellandrene	0.52 ± 0.07	0.44 ± 0.09
α-Terpinene	4.10 ± 0.13	4.22 ± 0.21
ϱ-Cimene	8.10 ± 0.15	8.11 ± 0.19
Limonene	1.01 ± 0.09	1.06 ± 0.12
1.8-Cineole	0.70 ± 0.09	0.54 ± 0.05
ß-Z-Ocimene	0.71 ± 0.08	0.72 ± 0.08
ß-E-Ocimene	0.32 ± 0.09	0.23 ± 0.09
γ-Terpinene	24.32 ± 0.36	25.11 ± 0.03
cis-Sabinene hydrate	0.82 ± 0.10	0.87 ± 0.07
Terpinolene	0.33 ± 0.09	0.34 ± 0.11
Linalool	0.71 ± 0.09	0.62 ± 0.08
Borneol	0.22 ± 0.10	0.31 ± 0.07
Terpinel-4-ol	1.32 ± 0.10	1.23 ± 0.12
α-Terpineol	0.42 ± 0.08	0.43 ± 0.07
Thymol methyl ethere	0.61 ± 0.08	0.62 ± 0.07
Carvacrol methyl ethere	2.82 ± 0.14	2.93 ± 0.16
Thymol	40.01 ± 0.45	40.03 ± 0.50
Carvacrol	1.52 ± 0.14	1.63 ± 0.19
ß-Caryophyllene	0.53 ± 0.10	0.55 ± 0.19
α-Humulene	0.32 ± 0.08	0.33 ± 0.07
γ-Muurolene	0.33 ± 0.05	0.28 ± 0.07
ß-Bisabolene	1.41 ± 0.11	1.52 ± 0.11
γ-Cadinene	0.32 ± 0.08	0.33 ± 0.06
Δ-Cadinene	0.53 ± 0.08	0.52 ± 0.09

Table S2. Effect of irrigation water on chemical composition of oregano essential oil. Average values (± standard deviation) are shown ($n = 6$).

Compound	Freshwater	Treated wastewater	Rainfed (control)
α-Thujene	2.10 ± 0.12	2.11 ± 0.15	2.08 ± 0.15
α-Pinene	1.10 ± 0.12	0.91 ± 0.10	0.92 ± 0.10
Sabinene	0.21 ± 0.05	0.20 ± 0.05	0.24 ± 0.05
ß-Pinene	0.62 ± 0.08	0.60 ± 0.05	0.60 ± 0.08
ß-Myrcene	3.01 ± 0.08	3.02 ± 0.17	3.01 ± 0.12
α-Phellandrene	0.51 ± 0.05	0.41 ± 0.08	0.41 ± 0.08
α-Terpinene	4.21 ± 0.21	4.11 ± 0.19	4.09 ± 0.17
ϱ-Cimene	8.02 ± 0.14	8.10 ± 0.22	8.03 ± 0.19
Limonene	1.12 ± 0.08	0.91 ± 0.12	0.93 ± 0.12
1.8-Cineole	0.60 ± 0.08	0.64 ± 0.11	0.61 ± 0.11
ß-Z-Ocimene	0.72 ± 0.07	0.71 ± 0.10	0.71 ± 0.10
ß-E-Ocimene	0.30 ± 0.10	0.32 ± 0.10	0.31 ± 0.10
γ-Terpinene	24.40 ± 0.33	24.31 ± 0.54	24.45 ± 0.54
cis-Sabinene hydrate	0.81 ± 0.05	0.82 ± 0.08	0.79 ± 0.08
Terpinolene	0.30 ± 0.12	0.32 ± 0.10	0.30 ± 0.10
Linalool	0.70 ± 0.06	0.60 ± 0.01	0.62 ± 0.06
Borneol	0.22 ± 0.12	0.21 ± 0.08	0.23 ± 0.08
Terpinel-4-ol	1.30 ± 0.10	1.32 ± 0.13	1.34 ± 0.13
α-Terpineol	0.41 ± 0.08	0.41 ± 0.08	0.42 ± 0.08
Thymol methyl ethere	0.62 ± 0.10	0.61 ± 0.09	0.62 ± 0.09
Carvacrol methyl ethere	2.80 ± 0.15	2.94 ± 0.19	2.90 ± 0.19
Thymol	39.90 ± 0.63	40.01 ± 0.26	40.64 ± 0.25
Carvacrol	1.50 ± 0.15	1.62 ± 0.16	1.54 ± 0.11
ß-Caryophyllene	0.51 ± 0.09	0.62 ± 0.10	0.63 ± 0.10
α-Humulene	0.31 ± 0.05	0.41 ± 0.05	0.38 ± 0.05
γ-Muurolene	0.30 ± 0.05	0.21 ± 0.05	0.22 ± 0.05
ß-Bisabolene	1.41 ± 0.14	1.51 ± 0.15	1.52 ± 0.15
γ-Cadinene	0.30 ± 0.08	0.31 ± 0.08	0.32 ± 0.08
Δ-Cadinene	0.51 ± 0.12	0.52 ± 0.08	0.51 ± 0.08
